# Blood Cadmium and Estimated Glomerular Filtration Rate in Korean Adults

**DOI:** 10.1289/ehp.1003054

**Published:** 2011-08-11

**Authors:** Young Hwangbo, Virginia M. Weaver, Maria Tellez-Plaza, Eliseo Guallar, Byung-Kook Lee, Ana Navas-Acien

**Affiliations:** 1 Department of Environmental Health Sciences, Johns Hopkins Bloomberg School of Public Health, Baltimore, Maryland, USA;; 2 Department of Preventive Medicine, Soonchunhyang University School of Medicine, Asan, Choongnam, Korea; 3 Department of Medicine, Johns Hopkins Medical Institutions, Baltimore, Maryland, USA; 4 Department of Epidemiology and Welch Center for Prevention, Epidemiology and Clinical Research, Johns Hopkins Bloomberg School of Public Health, Baltimore, Maryland, USA; 5 Department of Cardiovascular Epidemiology and Population Genetics, Centro Nacional de Investigaciones Cardiovasculares, Madrid, Spain; 6 Institute of Environmental and Occupational Medicine, Soonchunhyang University School of Medicine, Asan, Choongnam, Korea

**Keywords:** cadmium, chronic kidney disease, glomerular filtration rate, Korean, survey

## Abstract

Background: Cadmium is a nephrotoxicant at high exposure levels. Few studies have evaluated the role of cadmium in kidney function at low-exposure levels.

Objective: We evaluated the association of blood cadmium with estimated glomerular filtration rate (eGFR) in the Korean adult population.

Methods: We evaluated 1,909 adults ≥ 20 years of age who participated in the 2005 Korean National Health and Nutrition Examination Survey and had blood cadmium determinations. eGFR was calculated using the Modification of Diet in Renal Disease equation.

Results: Blood cadmium geometric means were 1.57 μg/L for men and 1.49 μg/L for women. The difference in eGFR levels that compared participants in the highest versus lowest cadmium tertiles, after multivariable adjustment, was –1.85 [95% confidence interval (CI): –3.55, –0.16] mL/min per 1.73 m^2^ in women and 0.67 (–1.16, 2.50) mL/min per 1.73 m^2^ in men. Among men, the association between blood cadmium and eGFR was modified by blood lead levels (*p*-value for interaction = 0.048). The fully adjusted differences in eGFR levels for a 2-fold increase in blood cadmium levels were –1.14 (–3.35, 1.07) and 1.84 (0.54, 3.14) mL/min per 1.73 m^2^ in men with blood lead levels below and above the median (2.75 μg/dL), respectively.

Conclusion: Elevated blood cadmium levels were associated with lower eGFR in women, which supports the role of cadmium as a risk factor for chronic kidney disease. In men, there was no overall association, although elevated blood cadmium levels were associated with higher eGFR levels in men with high blood lead levels and nonstatistically associated with lower eGFR levels in men with low blood lead levels.

Chronic kidney disease (CKD) is an increasing public health problem worldwide, resulting in substantial morbidity and mortality related to cardiovascular disease and end-stage renal disease (Coresh et al. 2007; Meguid El Nahas and Bello 2005). Established risk factors for CKD include diabetes (Triyanti et al. 2008), hypertension (Hajjar and Kotchen 2003; Rossert and Wauters 2002), and metabolic syndrome (Ryu et al. 2009). In addition, environmental exposures, including toxic metals, are potentially important CKD risk factors that have been understudied in general populations (Muntner et al. 2003; Navas-Acien et al. 2009). Cadmium, a widely distributed metal in the environment, is a well-known nephrotoxicant at high levels of exposure (Järup et al. 1995; Nordberg et al. 2007). Recent epidemiologic evidence from the United States and other countries also supports cadmium-related nephrotoxicity at relatively low levels of exposure (Åkesson et al. 2005; Chen et al. 2006; Ferraro et al. 2010; Hellström et al. 2001; Jin et al. 2004; Navas-Acien et al. 2009; Thomas et al. 2009). Data on associations between low-level cadmium exposure and estimated glomerular filtration rate (eGFR), however, remain scarce.

The objective of this study was to evaluate associations of blood cadmium with eGFR in South Korean adults who participated in the 2005 Korean National Health and Nutrition Examination Survey (KNHANES III). This is a particularly relevant population because almost 7% of South Korean adults have eGFR < 60 mL/min per 1.73 m^2^ based on the Modification of Diet Renal Disease (MDRD) Study equation (Jang et al. 2010), and cadmium exposure may be more common than in other countries because of soil contamination from metal mining and smelting processes in South Korea (Hong et al. 2009; Jung 2008; National Institute of Agricultural Science and Technology 1997). Major sources of cadmium in the general population of Korea include dietary intake (e.g., rice, vegetables) due to uptake from contaminated soil, and smoking (Eunha et al. 2006; Lee et al. 1986; Moon et al. 1995). Indeed, blood cadmium levels measured in a representative sample of South Korean adults were markedly higher than levels measured in a representative sample of U.S. adults (Kim and Lee 2011).

## Materials and Methods

*Study population.* KNHANES III was conducted by the Korean Ministry of Health and Welfare in 2005 using a stratified, multistage, probability cluster sampling design to select a representative sample of the South Korean population. Based on the 2000 National Census Registry, KNHANES randomly selected 600 sampling units for health interviews and 200 of those for health examinations, from 246,097 primary sampling units, each consisting of approximately 60 households. The participation rate for the health examination was 70.2% (7,597 participants). Among 5,501 participants ≥ 20 years of age, KNHANES randomly selected 10–12 subjects from each sampling unit while maintaining a uniform distribution across gender and five age groups (20–29, 30–39, 40–49, 50–59, ≥ 60 years) to yield a total of 1,998 subjects to measure lead, cadmium, and mercury in whole blood. We excluded 12 pregnant women, 3 participants missing serum creatinine, 20 participants missing height, weight, or other biochemistry variables, and 54 participants missing questionnaire responses for tobacco or alcohol consumption, leaving 1,909 participants for this study. The study design was approved by the Institutional Review Board of the Soonchunhyang University, College of Medicine. All participants provided written, informed consent.

*Blood cadmium and lead.* Blood samples for metal analysis were drawn into trace-metal–free tubes. The Seoul Medical Science Institute measured cadmium and lead in whole blood using graphite furnace atomic absorption spectrometry (model SpectrAA-800, with Zeeman correction; Varian Instruments, Agilent Technologies, Mulgrave, Australia). The limit of detection was 0.30 μg/L for blood cadmium and 0.23 μg/dL for blood lead. A total of 8 (0.4%) and 5 (0.3%) participants had blood cadmium and lead levels, respectively, below the limit of detection. For those participants, we imputed a level equal to the limit of detection divided by the square root of 2 (Hornung and Reed 1990). For internal quality assurance and control, standard reference materials were obtained from Bio-Rad (Lyphochek® Whole Blood Metals Control; Bio-Rad Laboratories, Hercules, CA, USA). The interassay coefficients of variation ranged from 10.0% to 21.1% for three blood cadmium samples with levels 4.30, 1.11, and 0.37 μg/L and from 7.6% to 8.9% for three blood lead samples with levels 48.0, 26.0, and 8.5 μg/dL. With respect to external quality programs, the Seoul Medical Science Institute has a certified license from the Korean Ministry of Labor as a designated laboratory for heavy metal analyses and has passed the German External Quality Assessment Scheme operated by Friedrich-Alexander-Universität Erlangen-Nürnberg. For KNHANES, the Korean Institute of Science and Technology randomly selected and reanalyzed 30 blood samples using graphite furnace atomic absorption spectrometry. The interlaboratory reliability for cadmium (*r* = 0.73) was lower compared with lead (*r* = 0.91).

*Measures of kidney outcomes.* KNHANES measured serum creatinine with a kinetic Jaffé method using an autoanalyzer (model 747; Hitachi, Tokyo, Japan). We used the MDRD Study equation to estimate eGFR, as an indicator of glomerular function: eGFR (milliliters per minute per 1.73 m^2^) = 186.3 × (serum creatinine)^–1.154^ × (age)^–0.203^ (× 0.742 if the individual was female). The number of participants with eGFR levels < 60 mL/min per 1.73 m^2^ was relatively small (36 men and 84 women, corresponding to weighted prevalences for the Korean population of 3.3% and 8.2%, respectively). For this study, we categorized participants as having reduced eGFR if their eGFR levels were below the 25th percentile using sex-specific cutoffs (< 74.7 mL/min per 1.73 m^2^ for men and < 65.4 mL/min per 1.73 m^2^ for women).

*Other variables.* Information on age, sex, education, menopause status, smoking, and alcohol consumption was based on self-report. Urbanization was categorized as metropolitan (superior administrative district that includes Seoul and six other large cities), urban (other cities in Korea), and rural (other localities). Body mass index (BMI) was calculated by dividing measured weight in kilograms by squared measured height in meters. Blood pressure was measured three times after 5 min of rest during the health examination, using a standard mercury sphygmomanometer (Baumanometer; W.A. Baum Co. Inc., New York, NY, USA). The last two measurements were averaged and used to estimate the prevalence of hypertension, which was defined as mean systolic blood pressure ≥ 140 mmHg, mean diastolic blood pressure ≥ 90 mmHg, or self-reported antihypertensive medication use. Diabetes mellitus was defined as fasting glucose ≥ 126 mg/dL, nonfasting glucose ≥ 200 mg/dL, or self-reported medication use (insulin and oral hypoglycemic treatment).

*Statistical analysis.* We performed all statistical analyses using the svy commands in STATA (version 11; StataCorp, College Station, TX, USA) to account for the complex sampling design and weights in KNHANES 2005 and to obtain results that are representative of the South Korean population. The distributions of blood cadmium and lead were right skewed and were log-transformed for the analyses. We categorized blood cadmium levels in tertiles, based on weighted distributions in the whole study sample. Participant characteristics by cadmium tertiles were compared using *p*-values for trend based on the Wald test. The statistical significance level was set at α = 0.05. All statistical analyses were two sided.

We observed significant differences by sex in the association between blood cadmium and eGFR (*p*-value for interaction = 0.004). Consequently, we conducted all analyses separately for men and women. Differences [95% confidence intervals (CIs)] in eGFR levels were estimated comparing the highest and the second highest tertiles to the lowest tertile (baseline) of blood cadmium using linear regression (i.e., tertiles 2 and 3 vs. tertile 1). We also used logistic regression to examine odds ratios of reduced eGFR (< 74.7 mL/min per 1.73 m^2^ in men, < 65.4 mL/min per 1.73 m^2^ in women) associated with increased blood cadmium levels. Statistical models were progressively adjusted for age (continuous), education (< high school, high school, > high school), living region (metropolitan/urban/rural), BMI (continuous), smoking status (never/former/current), alcohol intake (never/former/current), and blood lead (log transformed). We observed no substantial changes with further adjustments for hypertension and diabetes status, and these variables were not retained in the final models.

We estimated fully adjusted differences in eGFR associated with a 2-fold increase in blood cadmium levels by sex and in subgroups defined by age (< 40, 40–60, > 60 years), BMI (< 25, ≥ 25 kg/m^2^), education (< high school, high school, > high school), living region (metropolitan, urban, rural), smoking status (never, former, current), alcohol intake (never, former, current), and blood lead levels (below and above the median, 2.75 μg/dL). We tested for statistical interactions by including the product of log-transformed cadmium and each covariate in the regression models.

## Results

*Participant characteristics.* Mean eGFR was 81.5 mL/min per 1.73 m^2^ in men and 74.4 mL/min per 1.73 m^2^ in women. Men and women with reduced eGFR were older, less likely to have more than a high school education, and more likely to have hypertension and diabetes mellitus (Table 1). Women but not men with reduced eGFR were more likely to have higher blood cadmium and lead levels.

**Table 1:** Participant characteristics by eGFR status and sex, KNHANES 2005.

Reduced eGFR*^a^*		
Males			Females		
Characteristic	No (*n* = 693)	Yes (*n* = 262)	No (*n* = 695)	Yes (*n* = 259)
Age (years)		40.1 ± 0.6		51.0 ± 1.1		40.3 ± 0.7		55.1 ± 0.7
Postmenopause*a*						25.1 (2.2)		62.6 (3.4)
Education								
< High school		18.4 (1.8)		26.9 (3.0)		29.1 (2.0)		57.4 (3.1)
High school		46.4 (2.2)		43.9 (3.8)		42.7 (2.2)		34.0 (2.7)
> High school		35.2 (2.0)		29.3 (3.5)		28.2 (2.1)		8.6 (1.8)
Region								
Metropolitan		40.2 (1.9)		50.1 (4.0)		51.1 (1.9)		44.3 (4.3)
Urban		39.7 (2.1)		35.3 (4.0)		31.5 (1.7)		31.8 (4.1)
Rural		20.0 (1.5)		14.6 (3.0)		17.4 (1.6)		23.9 (3.9)
BMI (kg/m^2^)		23.9 (0.2)		24.5 (0.2)		23.2 (0.1)		24.2 (0.2)
Smoking status								
Never		17.3 (1.8)		13.9 (2.2)		88.7 (1.6)		89.5 (2.2)
Former		31.2 (2.3)		41.4 (3.0)		6.0 (1.2)		3.7 (1.3)
Current		51.6 (2.5)		44.6 (3.1)		5.3 (1.1)		6.8 (1.8)
Alcohol intake								
Never		3.5 (0.7)		9.4 (2.3)		14.4 (1.3)		23.3 (3.0)
Former		6.5 (1.1)		10.5 (2.0)		10.8 (1.4)		14.0 (2.4)
Current		90.0 (1.4)		80.1 (2.8)		74.7 (1.9)		62.7 (3.7)
Hypertension		22.0 (1.9)		39.2 (3.3)		13.5 (1.3)		36.8 (3.3)
Diabetes mellitus		6.3 (1.0)		9.8 (1.9)		4.2 (0.8)		8.3 (1.8)
Blood cadmium (μg/L)*b*		1.59 (1.51, 1.67)		1.52 (1.44, 1.62)		1.45 (1.39, 1.51)		1.65 (1.56, 1.74)
Blood lead (μg/dL)*b*		3.00 (2.85, 3.15)		2.93 (2.7, 3.18)		2.16 (2.04, 2.29)		2.80 (2.57, 3.06)
Values are mean ± SE or percentage (SE), unless otherwise noted. All values are weighted. **a**There were 88 women with missing data for menopausal status (*n* = 866). **b**Geometric mean (95%CI).								

The overall geometric mean of blood cadmium was 1.57 μg/L in men and 1.49 μg/L in women (data not shown). The overall geometric mean of blood lead was 2.98 μg/dL in men and 2.31 μg/dL in women (data not shown). Blood cadmium and lead were higher in men without reduced eGFR and in women with reduced eGFR, compared with men with reduced eGFR and women without reduced eGFR, respectively (Table 1). The weighted Pearson’s correlation coefficient between blood cadmium and lead levels was –0.05 (*p* = 0.12) in men and –0.01 (*p* = 0.87) in women (data not shown). Men who lived in the metropolitan city or who were former smokers or current alcohol drinkers had lower blood cadmium levels than did other men (*p*-value for trend < 0.05). For women, increasing age, lower education, and lower eGFR were associated with higher blood cadmium levels, whereas high school education was associated with lower blood cadmium levels (*p*-value for trend < 0.05). Increasing blood cadmium levels were associated with the prevalence of hypertension in both men and women (Table 2).

**Table 2:** Participant characteristics by sex and blood cadmium level.

Men						Women					
Blood cadmium tertile			Blood cadmium tertile		
Characteristic	Total	< 1.29 μg/L	1.29–1.88	> 1.88 μg/L	*p*-Value for trend*a *	Total	< 1.29 μg/L	1.29–1.88	> 1.88 μg/L	*p*-Value for trend*a*
*n* (%)		955 (100)		293 (30.3)	335 (34.9)	327 (34.8)			954 (100)		341 (36.4)	308 (31.9)	305 (31.7)	
Age (years)*b*				42.2 (40.4, 44.0)	43.1 (41.2, 45.0)	43.1 (41.1, 45.1)	0.74				42.0 (40.2, 43.7)	43.5 (41.5, 45.6)	46.9 (45.2, 48.7)	< 0.01
Postmenopause (%)*c*				—	—	—	—		332		31.2	31.9	36.9	0.06
Education (%)														
< High school		235		25.0	35.7	39.3	0.11		384		30.7	29.1	40.2	< 0.01
High school		409		28.6	36.7	34.7	0.62		374		39.6	34.5	25.9	< 0.01
> High school		311		35.8	32.0	32.2	0.09		196		40.0	31.5	28.5	0.51
Region (%)														
Metropolitan		356		36.1	30.9	33.0	< 0.05		413		37.1	32.6	30.3	0.72
Urban		391		27.2	38.9	33.9	0.25		332		37.5	30.1	32.4	0.80
Rural		208		23.5	35.8	40.7	0.27		209		32.9	32.7	34.4	0.61
BMI [ ≥ 25 kg/m^2^ (%)]		381		30.7	36.9	32.4	0.60		298		31.3	36.0	32.7	0.12
Smoking status (%)														
Never		138		28.0	36.2	35.8	0.82		861		37.4	31.9	30.7	0.24
Former		343		35.7	34.3	30.0	< 0.05		42		34.9	28.6	36.5	0.81
Current		474		27.4	34.9	37.7	0.12		51		23.1	33.6	43.3	0.11
Alcohol intake (%)														
Never		50		22.2	32.2	45.6	0.26		177		29.5	36.8	33.7	0.12
Former		86		23.3	30.6	46.1	0.17		112		35.6	32.9	31.5	0.97
Current		819		31.4	35.4	33.2	< 0.05		665		38.2	30.5	31.3	0.25
Blood lead (μg/dL)*d*		955		3.06 (2.89, 3.24)	3.00 (2.83, 3.18)	2.88 (2.64, 3.14)	0.51		954		2.35 (2.19, 2.53)	2.36 (2.18, 2.55)	2.22 (2.04, 2.41)	0.43
Diabetes mellitus (%)		87		40.6	31.0	28.4	0.17		54		34.3	22.6	43.1	0.17
Hypertension (%)		273		22.6	33.8	43.6	< 0.01		193		26.9	30.2	42.9	< 0.01
eGFR (mL/min/1.73 m^2^)*e *		955		80.1 (78.4, 81.8)	81.4 (80.0, 82.8)	80.3 (78.4, 82.2)	0.41		954		75.3 (73.5, 77.1)	73.1 (71.7, 74.5)	72.0 (70.5, 73.5)	< 0.01
Reduced eGFR (%)		262		33.4	34.1	32.5	0.56		259		28.7	32.1	39.2	< 0.01
—, data not available. All values are weighted. Tertiles are based on the weighted distribution in the whole study sample. **a***p*-Value for trend estimated based on the Wald test. **b**Mean (95% CI). **c**There were 88 women with missing menopause status (*n* = 866). **d**Geometric mean (95% CI). **e**eGFR < 74.7 mL/min per 1.73 m^2^ for men and eGFR < 65.4 mL/min per 1.73 m^2^ for women; mean eGFR (95% CI).														

*Blood cadmium levels and eGFR.* After adjustment for sociodemographic factors, CKD risk factors and blood lead levels, the difference in eGFR levels comparing participants in the highest versus the lowest tertiles of blood cadmium was 0.67 (95% CI: –1.16, 2.50) mL/min per 1.73 m^2^ in men and –1.85 (–3.55, –0.16) mL/min per 1.73 m^2^ in women (Table 3, model 3). Similar results were found for reduced eGFR (Table 4). Fully adjusted odds ratio for reduced eGFR (< 74.6 mL/min per 1.73 m^2^ in men and < 65.4 mL/min per 1.73 m^2^ in women) comparing the highest versus lowest blood cadmium tertiles was 0.75 (95% CI: 0.49, 1.15) in men and 1.62 (1.00, 2.62) in women.

**Table 3:** Difference (95% CI) in eGFR levels (mL/min per 1.73m^2^) by blood cadmium tertile.

Blood cadmium level (μg/L)	*n*	Model 1*a*	Model 2*b*	Model 3*c*
Men								
< 1.29		293		0 (Reference)		0 (Reference)		0 (Reference)
1.29–1.88		335		1.24 (–0.83, 3.31)		1.32 (–0.44, 3.08)		1.38 (–0.37, 3.13)
> 1.88		327		0.50 (–1.70, 2.71)		0.42 (–1.41, 2.25)		0.67 (–1.16, 2.50)
Women								
< 1.29		341		0 (Reference)		0 (Reference)		0 (Reference)
1.29–1.88		308		–2.59 (–4.71, –0.48)		–1.92 (–3.73, –0.11)		–1.94 (–3.73, –0.15)
> 1.88		305		–3.58 (–5.65, –1.52)		–1.56 (–3.33, 0.20)		–1.85 (–3.55, –0.16)
Tertiles were based on the weighted distribution in the whole study sample. **a**Not adjusted (crude). **b**Adjusted for age, education, region (metropolitan, urban, rural), and BMI. **c**Included variables in model 2 and smoking status (never, former, current), alcohol intake (never, former, current), and blood lead (log micrograms per deciliter).								

**Table 4:** Odds ratios (95% CIs) for reduced eGFR*a* by blood cadmium level.

Blood cadmium level (μg/L)	Cases/noncases	Model 1*b*	Model 2*c*	Model 3*d*
Men								
< 1.29		88/205		1 (Reference)		1 (Reference)		1 (Reference)
1.29–1.88		87/248		0.85 (0.56, 1.27)		0.76 (0.48, 1.20)		0.76 (0.48, 1.21)
> 1.88		87/240		0.80 (0.53, 1.21)		0.80 (0.52, 1.24)		0.75 (0.49, 1.15)
Women								
< 1.29		75/266		1 (Reference)		1 (Reference)		1 (Reference)
1.29–1.88		86/222		1.37 (0.94, 1.99)		1.31 (0.82, 2.07)		1.30 (0.82, 2.08)
> 1.88		98/207		1.82 (1.25, 2.67)		1.49 (0.92, 2.40)		1.62 (1.00, 2.62)
Tertiles are based on the weighted distribution in the whole study sample. **a**eGFR < 74.7 mL/min per 1.73 m^2^ for men and eGFR < 65.4 mL/min per 1.73 m^2^ for women. **b**Not adjusted (crude). **c**Adjusted for age, education, region (metropolitan, urban, rural), BMI. **d**Included variables in model 2 and smoking status (never, former, current), alcohol intake (never, former, current), and blood lead (log micrograms per deciliter).								

*Subgroup analysis.* Among women, the association between blood cadmium and eGFR levels was consistent by all participant characteristics evaluated (Table 5). Among men, the association between blood cadmium and eGFR was modified by blood lead levels (*p*-value for interaction = 0.048) and education (*p*-value for interaction = 0.026), and maybe by alcohol intake (*p*-value for interaction = 0.052). The fully adjusted difference in eGFR levels for a 2-fold increase in blood cadmium levels in men was –1.14 (95% CI: –3.35, 1.07) mL/min per 1.73 m^2^ for blood lead ≤ 2.75 μg/dL and 1.84 (0.54, 3.14) mL/min per 1.73 m^2^ for blood lead > 2.75 μg/dL (Table 5). Similarly, the difference in eGFR levels comparing the highest to the lowest cadmium tertile for men with blood lead levels below and above 2.75 μg/dL were –2.01 (95% CI: –5.43, 1.41) and 2.47 (0.41, 4.53) mL/min per 1.73 m^2^, respectively. The odds ratio for eGFR < 74.7 mL/min per 1.73 m^2^ comparing the highest to the lowest cadmium tertile for men with blood lead levels below and above 2.75 μg/dL were 1.30 (95% CI: 0.64, 2.64) and 0.56 (0.33, 0.93), respectively. By education, blood cadmium levels were positively associated with eGFR levels in men with more than a high school education but not in men with a high school education or less. By alcohol intake, blood cadmium levels were positively associated with eGFR levels, but effect modification was borderline statistically significant.

**Table 5:** Difference (95% CI) in eGFR levels (milliliters per minute per 1.73 m^2^) comparing a 2-fold increase in blood cadmium by sex and other participant characteristics.

Men					Women				
Subgroup	*n*	Difference (95% CI)	*p*-Value for interaction*a*	*n*	Difference (95% CI)	*p*-Value for interaction*a*
Overall		955		0.68 (–0.47, 1.84)				954		–1.51 (–2.61, –0.40)		
Age (years)												
< 40		337		0.78 (–0.90, 2.46)		0.61		365		–2.46 (–4.16, –0.77)		0.06
40–60		456		0.72 (–1.19, 2.63)				411		–1.20 (–2.85, 0.46)		
> 60		162		–1.53 (–5.07, 2.01)				178		0.11 (–2.40, 2.62)		
BMI (kg/m^2^)												
< 25		574		0.36 (–1.25, 1.96)		0.21		656		–1.81 (–3.10, –0.53)		0.65
≥ 25		381		1.11 (–0.66, 2.88)				298		–1.08 (–2.91, 0.75)		
Education												
< High school		235		–0.51 (–3.30, 2.28)		0.026		384		–0.30 (–2.04, 1.45)		0.13
High school		409		0.09 (–1.78, 1.96)				374		–2.38 (–3.88, –0.87)		
> High school		311		2.27 (0.88, 3.66)				196		–2.18 (–4.27, –0.09)		
Region												
Metropolitan		356		0.53 (–1.21, 2.27)		0.51		413		–1.30 (–2.77, 0.16)		0.87
Urban		391		2.34 (0.40, 4.27)				332		–2.46 (–4.42, –0.50)		
Rural		208		–2.45 (–5.42, 0.51)				209		–0.87 (–3.65, 1.91)		
Smoking												
Never		138		2.61 (0.25, 4.97)		0.18		861		–1.35 (–2.50, –0.21)		0.83
Former		343		0.14 (–2.07, 2.36)				42		–6.28 (–10.77, –1.78)		
Current		474		0.38 (–1.23, 2.00)				51		0.41 (–3.12, 3.95)		
Alcohol intake												
Never		50		–6.78 (–14.61, 1.04)		0.052		177		–1.34 (–4.04, 1.35)		0.63
Former		86		0.58 (–3.93, 5.09)				112		–3.07 (–5.87, –0.27)		
Current		819		1.19 (0.03, 2.35)				665		–1.51 (–2.66, –0.35)		
Blood lead (μg/dL)												
≤ 2.75		350		–1.14 (–3.35, 1.07)		0.048		567		–1.57 (–3.16, 0.01)		0.78
> 2.75		605		1.84 (0.54, 3.14)				387		–1.36 (–2.70, –0.01)		
Adjustment included age, BMI, education level, region (metropolitan, urban, rural), smoking (never, former, current), drinking (never, former, current), and blood lead (log transformed). **a***p*-Value for interaction was estimated including an interaction term between log-transformed blood cadmium and the corresponding group of categories.												

## Discussion

In a representative sample of Korean adults who participated in KNHANES III in 2005, elevated blood cadmium levels were associated with lower eGFR levels in women but not in men. In women, a 2-fold increase in blood cadmium was associated with a statistically significant 1.51 mL/min per 1.73 m^2^ decrease in eGFR levels, with no difference by blood lead levels. In men, a 2-fold increase in blood cadmium was associated with a nonstatistically significant 0.68 mL/min per 1.73 m^2^ increase in eGFR levels, with possible effect modification by lead levels: in men with blood lead ≤ 2.75 μg/dL, a 2-fold increase in blood cadmium was associated with a non-statistically significant 1.14 mL/min per 1.73 m^2^ decrease in eGFR levels. In contrast, in men with blood lead > 2.75 μg/dL, a 2-fold increase in blood cadmium was associated with a statistically significant 1.84 mL/min per 1.73 m^2^ increase in eGFR levels. These findings among men need to be interpreted cautiously as they were the result of a post hoc analysis with no *a priori* hypothesis for effect modification of the relation between cadmium and eGFR by lead levels.

Cadmium exposure in Asia (China, Japan, Korea) is generally higher than in Europe and the United States, because of the high number of cadmium–polluting industries and high intake of rice and vegetables grown locally in cadmium-contaminated soil (Nordberg et al. 2007). In KNHANES, blood cadmium levels were comparable to those found in the Japanese general population (geometric mean, 1.76 μg/L) (Ikeda et al. 2000) and around four times higher than blood cadmium levels measured in the U.S. general population (geometric mean, 0.41 μg/L) (Navas-Acien et al. 2009) and in Swedish women (median, 0.38 μg/L) (Åkesson et al. 2005). Also, although in the United States and other countries cadmium and lead are moderately correlated (Åkesson et al. 2005; Navas-Acien et al. 2009), in KNHANES cadmium and lead were not correlated, indicating that the sources of exposure for cadmium and lead could be different. For cadmium, diet is thought to be the main source of exposure for the general Korean population, whereas for lead the main source is thought to be ambient air (Eunha et al. 2006; Moon et al. 1995). In Korea, rice and barley samples from locations near mines or affected by mine drainage have shown significantly higher cadmium levels compared with samples from control areas (Kim et al. 2008). High consumption of cadmium-contaminated rice may explain higher cadmium levels in Korean adults compared with populations characterized by lower rice consumption (Kim and Lee 2011). Dietary sources of cadmium, however, are unlikely to explain the similar cadmium levels in men and women in KNHANES, compared with most other populations in which women tend to have higher cadmium levels. Higher smoking rates among men than among women could also result in more comparable blood cadmium levels in men and women than in other studies. Information on occupational exposure or job titles, unfortunately, was not available, and their relationship with cadmium levels in men and women could not be evaluated.

Early cadmium-induced kidney damage is characterized by proximal tubule effects manifested by increased urinary excretion of low-molecular-weight proteins such as β2-microglobulin (Nordberg et al. 2007). Tubular damage may progress to glomerular damage and eventually to kidney failure in the setting of high-level cadmium exposure (Järup et al. 1998; Jin et al. 2004). In animal studies, cadmium-treated rats showed enhanced glomerular filtration of proteins compared with controls, although cadmium levels used in these experiments were markedly higher compared with human populations (Bernard et al. 1992; Cardenas et al. 1992). Recently, a number of observational studies have shown adverse kidney effects at much lower cadmium levels (Åkesson et al. 2005; Hellström et al. 2001; Jin et al. 2004; Navas-Acien et al. 2009; Thomas et al. 2009). In the U.S. general population, increasing blood cadmium levels were associated with increased prevalences of albuminuria and eGFR < 60 mL/min per 1.73 m^2^ (Navas-Acien et al. 2009).

In our study, despite similar blood cadmium levels in men and women, associations between blood cadmium and eGFR differed markedly by sex. In women, higher blood cadmium levels were associated with lower eGFR, consistent with other cross-sectional studies on low-level cadmium exposure and clinical kidney disease measures (Åkesson et al. 2005; Navas-Acien et al. 2009) and supportive of cadmium as a CKD risk factor. The association in women was independent of blood lead levels. Some reports suggest that cadmium-related renal effects may be greater in women compared with men. For example, itai-itai disease, a combination of kidney disease and bone damage, affected mainly women in cadmium-polluted areas in the Jinzu River of Toyama prefecture, Japan (Aoshima 1987; Kjellström et al. 1977; Nomiyama 1980). Although cadmium intake differed slightly between men and women, Nishijo et al. (2004) found that urine levels of cadmium, β2-microglobulin, and amino acids were higher in women than in men. In Sweden, after adjusting for age and sex, Hellström et al. (2001) reported that the risk of renal replacement therapy was higher for cadmium-exposed participants than for unexposed participants and that the association between cadmium and renal replacement therapy was stronger among women than among men. Research in other populations and experimental models are needed to confirm heterogeneity of cadmium effects by sex on the kidney and to identify the responsible mechanisms.

For the men in our study, however, cadmium levels were not associated with eGFR overall. For men with blood lead levels at or below the median, the findings were consistent with those in women, although the association was not statistically significant. Among men with higher blood lead levels, elevated cadmium levels were associated with higher eGFR. The explanation for the positive association between blood cadmium and eGFR levels among men with high lead levels observed in this study remains unclear. Previously, three studies reported positive associations between urine cadmium levels and glomerular filtration measures, although differences by gender were not evaluated. In the Cadmibel study (*n* = 593), higher urine but not blood cadmium was prospectively associated with higher measured creatinine clearance in the 5-year follow-up, although the level of statistical significance was not reported (Hotz et al. 1999). In European children (*n* = 804), higher urine cadmium but not blood cadmium was cross-sectionally associated with lower serum creatinine after adjustment for other metals (de Burbure et al. 2006). No association was found for urine or blood cadmium with serum β2-microglobulin or serum cystatin C (de Burbure et al. 2006). In that study, higher blood lead (> 55 μg/L; mean, 78.4 μg/L) was associated with lower serum creatinine, higher β2-microglobulin, and higher serum cystatin C, a finding that was consistent with hyperfiltration, an increase in GFR. Recently, in 712 Korean lead workers (mean urine cadmium, 1.15 μg/g creatinine), higher urine cadmium levels were associated with higher estimated creatinine clearance, eGFR, and *N*-acetyl-β-d-glucosaminidase levels and with lower serum creatinine after adjustment for sociodemographic and CKD risk factors, urine creatinine, and blood and bone lead levels (Weaver et al. 2011). Differences in the association between urine cadmium and kidney measures by blood lead levels were not evaluated, although lead exposure levels in this occupational population (mean blood lead, 23.1 μg/dL) were substantially higher than in our study.

The interaction between lead and cadmium with eGFR was a post hoc finding and not based on an *a priori* hypothesis. One potential explanation for the positive association between cadmium and eGFR among men exposed to higher lead levels is hyperfiltration, a process in which GFR is initially increased but may be followed by subsequent declines in GFR, leading to CKD. Glomerular hyperfiltration has been associated with early diabetes, obesity, and high-protein diet and has been suggested in lead workers (Bosch et al. 1983, 1984; Chagnac et al. 2000, 2003; Christiansen 1985; Friedman 2004; Mogensen 1986; Roels et al. 1994; Tomaszewski et al. 2007; Weaver et al. 2003). In our study, we could not evaluate the effect of early diabetes on the association between blood cadmium and eGFR because of the lack of information on diabetes onset. However, we also observed stronger positive associations between cadmium and eGFR in men with higher BMI, although those differences were not statistically significant. In Korean lead workers, potential hyperfiltration associated with elevated blood lead was also found in younger workers, whereas in older workers blood lead levels were associated with worse renal function (Weaver et al. 2003).

There are several limitations to our study. First, we evaluated kidney function by estimating GFR instead of directly measuring it. GFR estimates may have greater inaccuracy in general populations compared with CKD patients (Stevens et al. 2006). Moreover, the MDRD equation was developed in an adult population from the United States (Beck et al. 1991; Kusek et al. 1993; Levey et al. 1999), and currently no correction is added based on Korean ethnicity. Compared with measured GFR using the ^99m^Tc-DTPA (technetium=99m-labeled diethylenetriamine pentaacetic acid) renal clearance method, precision and accuracy in 393 healthy Korean men and women were greater for the MDRD equation than for 100/serum creatinine, 24-hr urine creatinine clearance, the Cockcroft-Gault equation, or the African-American Study of Kidney Disease and Hypertension equation (Kang et al. 2005). Second, a single assessment of serum creatinine and blood cadmium was available. Urine cadmium, an established biomarker of chronic cadmium exposure, was not available. However, blood cadmium is a well-established biomarker of cadmium exposure. The half-life of blood cadmium is 3–4 months for a component reflecting ongoing exposure, with a longer component of approximately 10 years reflecting equilibrium with internal body burden (Järup and Åkesson 2009; Nordberg et al. 2007). Thus, after chronic low-level exposure typical of the environmental setting and relevant for this study, blood cadmium also indicates body burden. Although blood cadmium reflects both ongoing and past exposure, nondifferential measurement error in cadmium exposure could potentially bias the results toward the null. Moreover, laboratory determination of cadmium can be challenging, and we cannot completely discard laboratory error as a source of bias in this study. Third, it is difficult to determine the directionality of the association between blood cadmium and kidney function because this is a cross-sectional study. As a consequence, reverse causation cannot be excluded. Fourth, although the overall sample size was large, we found that the association of cadmium with eGFR differed by sex and possibly by lead levels in men. Because of the need to carry out subgroup analyses in men and women, our study may lack sufficient power to precisely estimate dose–response relationships in subgroups and could be subject to biases due to multiple comparisons. As a consequence, the sex and lead interactions described in this analysis will have to be confirmed in other studies. Strengths of the study include representation of the Korean population and the standardized study procedures.

## Conclusion

We found that elevated blood cadmium levels were associated with lower eGFR in women, supporting the role of cadmium as a CKD risk factor. In men, there was no overall association between cadmium levels and eGFR. In men with high blood lead levels, however, elevated blood cadmium levels were associated with higher eGFR levels. In men with low blood lead levels, elevated blood cadmium levels were non-statistically associated with lower eGFR levels. Relatively few studies have examined the impact of low-level environmental cadmium exposure on glomerular filtration in general populations. Evaluating the consistency of the different directionality of the association between cadmium and eGFR by lead levels in men, and elucidating potential mechanisms for this finding if confirmed, could have important implications for risk assessment of cadmium-related kidney effects. Additional prospective studies and experimental work in animal models are needed to fully understand the impact of low-level cadmium exposure on the kidney.
